# The cystic adventitial disease of the popliteal artery: from imaging to histopathology

**DOI:** 10.1590/1806-9282.20231482

**Published:** 2024-04-22

**Authors:** Jan Roman, František Jalůvka, Ilker Sengul, Demet Sengul, Tomáš Jonszta, Pavel Hurník, Anton Pelikán, Václav Procházka

**Affiliations:** 1 University Hospital Ostrava, Department of Surgery – Ostrava, Czech Republic.; 2 University of Ostrava, Faculty of Medicine, Department of Surgery – Ostrava, Czech Republic.; 3 Giresun University, Faculty of Medicine, Division of Endocrine Surgery – Giresun, Turkey.; 4 Giresun University, Faculty of Medicine, Department of Surgery – Giresun, Turkey.; 5 Giresun University, Faculty of Medicine, Department of Pathology – Giresun, Turkey.; 6 University Hospital Ostrava, Department of Radiology – Ostrava, Czech Republic.; 7 University Hospital Ostrava, Institute of Molecular and Clinical Pathology and Medical Genetics – Ostrava, Czech Republic.; 8 University of Ostrava, Institute of Molecular and Clinical Pathology and Medical Genetics, Faculty of Medicine – Ostrava, Czech Republic.; 9 Tomas Bata University in Zlin, Faculty of Humanities, Department of Health Care Sciences – Zlín, Czech Republic.

**Figure 1:**
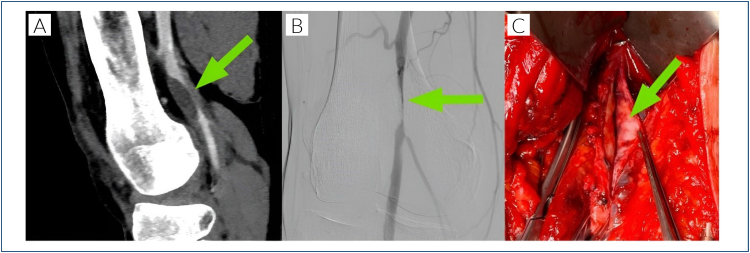
(A) A computed tomographic image showing the location of the stenosis at the P1 segment of the popliteal artery. (B) A digital subtraction angiography showing the eccentric, gradually rising stenosis. (C) An intraoperative image exhibiting the opened adventitial cyst with all gelatinous contents removed.

**Figure 2:**
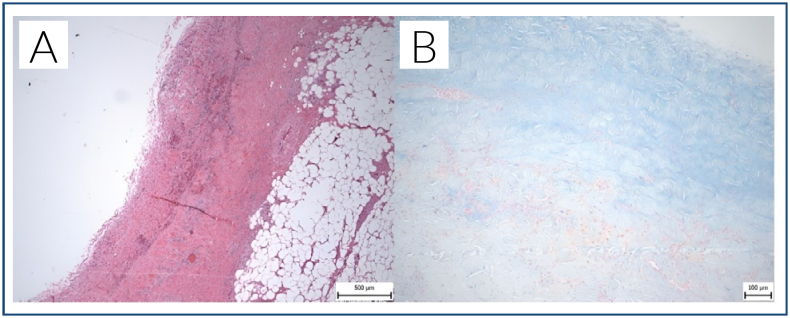
(A) Fibrous wall of a cystic lesion of the adventitia (hematoxylin-eosin; original magnification, 40×). (B) Alcian-positive mucoid masses diffusely permeating the wall (Alcian blue; original magnification, 40×).

Dear Editor,

Cystic adventitial disease (CAD) is a rare non-atherosclerotic vascular disease, presenting with claudication symptoms usually in young males without any risk factors. Even though many cases have been described, the precise cause of this disease remains unknown. The incidence of CAD is 1:1200 calf claudication cases^1^. The disease causes myxomatous cystic tumor growth in the adventitial layer of a blood vessel, effectively causing external compression of the lumen and slowing down the blood flow. It mostly involves arteries, typically in (but not limited to) the popliteal region. However, it can also affect veins. Even though this disease can only be observed in asymptomatic patients, the only definitive treatment relies on surgical removal or marsupialization of the cyst. The following vignette case describes the clinical factors, treatment, and response of an adult who presented with a CAD of the popliteal artery. A 41-year-old white male, treated only for Klinefelter syndrome with testosterone substitution, presented with 14 days of claudication pain in the right calf. He reported pain, which occurred after a walking distance of 50–100 m and was accompanied by cold and pins-and-needles sensations in the affected area, without any limitation to movement or sensitivity. However, no similar problems had been experienced beforehand, and on examination, a colder right leg below the knee was revealed, with no palpable pulse in the popliteal area or more distally, with a positive Homans sign. The rest of his physical examination was unremarkable, and laboratory findings were recognized in the physiological ranges. A Doppler ultrasonography demonstrated the signs of embolization in the area of popliteal artery trifurcation. Moreover, the proximal blood flow was not compromised with triphasic Doppler waveforms and 100–80 cm/s flow. However, distally from the popliteal artery division, no blood flow was notified. A well-developed collateral flow around the knee was also noticed and pathology of the deep vein system was detected. A computed tomographic (CT) angiography was performed, confirming the findings from the sonography and localized the area of obstruction to the P1 segment of the popliteal artery (Figure 1A). He was indicated for surgical embolectomy with the signs of popliteal artery embolization and, shortly after admission, an indirect embolectomy through the Fogarty catheters was performed. Of note, no embolus was removed. However, the arterial backflow was notified in normal values. Moreover, an intraoperative angiography was performed, exhibiting a residual narrowing of the P1 segment of the popliteal artery to 30–40% due to doubts about the quality of perfusion. Perioperatively, the clinical status of the affected limb was largely improved, therefore the surgical procedure was completed without any further interventions. Postoperatively, due to pathological stenosis in a young male, an investigation continued based on the clinical imaging studies. By using Doppler ultrasonography and antegrade digital subtraction angiography (DSA), the morphology of the stenosis was established as eccentric, gradually rising from the arterial wall, with a well-developed collateral arterial flow (Figure 1B). The flow of the contrast agent was slowed down. However, the morphology of the lower limb arterial tree was normal. As such, a diagnosis of CAD was suggested and the patient was scheduled for an early open surgical revision of the popliteal artery, which was performed 4 days later. During the surgery, in the prone position, using an S-shaped incision, a popliteal artery and a vein were identified. The subcutaneous tissue was highly fibrous with plenty of collateral vessels, and the preparation of both critical structures proved challenging. A 9,000 IU of heparin was administered for further embolization prevention and the procedure of vascular clamps was placed to prevent eventual unnecessary bleeding. Afterward, the adventitial layer of the popliteal artery was incised, revealing a small cavity containing gelatinous material similar to coagulated blood, albeit browner (Figure 1C). As there was no apparent communication with the arterial lumen, the applied clamps were released, without any signs of bleeding. In fine, the surgical incision was closed as usual with a placement of 10 French Redon drains after the total duration of the surgery of 160 min. Postoperatively, the 5700 IU of nadroparin was administered subcutaneously twice daily, together with 250 IU sulodexide per os also twice daily. No complications occurred during the postoperative period and his recovery was uneventful. A Doppler sonography was performed before discharge, exhibiting a normal triphasic flow in all parts of the arterial system of his lower extremity. During the follow-up, the patient was completely symptom-free at 1- and 3-month periods. Histopathological examination revealed a CAD consisting of an adventitial fibrous tissue with small blood vessels and discrete chronic inflammatory cellularization. Microscopically, the lumen of the cystic structure was not lined with an epithelium layer but filled with mucoid contents (Figure 2A). Histochemically, the wall of the cystic structure was permeated by the mucosubstances positivity in the Alcian blue staining (Figure 2B). The CAD was originally described in 1947 by Atkins and Key^2^. Even though many cases have been described, the precise pathophysiology remains unknown. Several theories have been proposed. However, none of them has been proven. The original proposition described mucinous degeneration of the adventitial layer^3^, but the theory of repeated trauma quickly replaced this theory^4^. As CAD can also affect other locations in the body and is present mostly in young patients with no history of trauma, an alternative theory of synovial involvement was soon proposed, which is based on the fact that vessels located near joints are usually affected. Of note, traumatic events affecting the joint capsule or a spontaneous migration of synovial cells cause the production of mucin along arterial or venous walls resulting in the development of cysts. Some authors also proposed the idea of the embryogenic origin of mucinous cells^5^. The disease mostly affects young people and males are affected approximately five times more often^6^, and commonly, less to no known risk factors are present for the aforementioned phenomenon. Affected patients typically present with claudication symptoms of various intensities and speeds of onset. Even though no other symptoms may be present, sometimes changes in peripheral pulse quality can be detected. These symptoms mostly result from the presence of arterial cysts (frequently in the popliteal region, in up to 85% of cases^7^). However, veins can also be rarely affected. In case of vein involvement, that condition may result in deep vein thrombosis. The first line diagnostic modality is Doppler ultrasound, which typically proves a reduction of blood flow distally from the affected area. Some sonographic signs are considered specific for CAD, such as cyst wall visualization, sonographic scimitar sign, and no vascularization of the cyst. However, due to the rarity of the disease, these findings usually need to be verified by CT angiography or DSA. To this end, these examinations are able to verify a typical shape of the stenosis (scimitar sign and hourglass shape of the lumen), which is usually gradual, with no post-stenotic dilation (as opposed to other forms of stenosis)^8^. Of note, magnetic resonance imaging is also a viable option, showing classical signs of cystic mass – hypointensity in T1- and hyperintensity in T2-weighted images. While some cases can be managed as "watch-and-wait" with regular check-ups, there were cases of spontaneous resolution of symptoms^9^. However, most are symptomatic and require treatment options. A step-up treatment can be rationally recommended. However, there is no strong evidence in the literature regarding the optimal treatment of CAD and the long-term prognosis of patients. As such, the first-line method is CT-guided aspiration of the cyst, which can be sometimes successful on its own. Notably, cases treated by the percutaneous intervention were also published, with variable outcomes. In case of recurrence, surgery is the method of choice in the management of this phenomenon. No high-quality comparison of various surgical methods is available, and the final treatment method depends on the experience of the surgeon. In English-language literature, the arterial region affected by a cyst is usually completely resected, with the affected region being replaced by a bypass procedure. This is successful in 94% of cases, and the overall long-term patency of grafts is high (80.9 months)^10^. In conclusion, peripheral arterial disorders^11,12^ remain the challenging issue of vascular surgery and vascular pathology. Anecdotally, the adventitial cystic disease of the popliteal artery might lead to claudication symptoms and mimicking symptoms of chronic limb Ischemia, mostly in younger, without risk factors. Available literature reveals that the affected arterial structure can easily be preserved. From a surgical perspective, physiological blood flow is usually restored, resulting in a complete resolution of symptoms, after surgical intervention. Postoperatively, the majority of cases remain symptom-free in the long term, and recurrences are typically described following interventional procedures. To this end, we once again emphasize that physicians should be vigilant for this rare entity during their daily routine in both out- and inpatient clinics.
